# Human beta-defensins induce APOBEC3G expression by interacting with chemokine receptors, protecting highly susceptible cells from HIV infection

**DOI:** 10.1186/1742-4690-9-S1-P8

**Published:** 2012-05-25

**Authors:** Alfredo Garzino-Demo, Mark K Lafferty, Jennifer Bharucha, Lingling Sun, Walter Royal, Suzanne Gartner, Wuyuan Lu

**Affiliations:** 1 Institute of Human Virology, U. Maryland School of Medicine, Baltimore, USA

## 

ß-defensins are antimicrobial peptides secreted by epithelial cells that can bind to cellular receptors. CCR2 and CCR6 are the two cellular receptors known to bind human ß-defensin (hBD) 2 and -3. Both of these receptors are of crucial relevance in HIV infection. CCR6 is expressed, often in concert with CCR5, on cells that are highly susceptible to HIV infection: memory T cells, Th_17_ cells, α4ß7^+^ cells, and defects in CD4^+^CCR6^+^ cells have been associated with faster AIDS progression. CCR2 is expressed on monocytes and macrophages, cells that are reservoirs of HIV infection and that are known to mediate central nervous system damage. Our studies show that hBD2, hBD3, and CCR6 ligand MIP-3α/CCL20 inhibit HIV infection via CCR6 by increasing expression of the antiviral protein APOBEC3G. This increase is due to a transcriptional mechanism mediated by intracellular signaling. hBD2 also inhibits HIV replication in macrophages that express CCR2. Our findings suggest novel therapeutic and preventive approaches that exploit CCR6 and CCR2-mediated intracellular signaling to inhibit HIV infection in highly susceptible cells.

